# Editorial: Regulation of Dynamic Changes and Remodeling Events During the Formation, Rescue and Regression of the Corpus Luteum

**DOI:** 10.3389/fendo.2020.00244

**Published:** 2020-04-24

**Authors:** Jens Vanselow, Lane K. Christenson, Joy L. Pate

**Affiliations:** ^1^Reproductive Biology, Leibniz Institute for Farm Animal Biology (FBN), Dummerstorf, Germany; ^2^Department of Molecular and Integrative Physiology, University of Kansas Medical Center, Kansas City, MO, United States; ^3^Department of Animal Science, Pennsylvania State University (PSU), University Park, PA, United States

**Keywords:** folliculo-luteal transition, granulosa cells, luteal cells, theca cells, apoptosis, necroptosis, autophagy, progesterone

Hormonal communication among the hypothalamus, pituitary, ovary, and uterus regulates the female reproductive cycle to provide fertilizable oocytes and a favorable environment for embryo implantation and fetal growth. In the ovary, a recurring sequence of cellular development, differentiation, cell survival and cell death, better known as folliculogenesis, ovulation, luteinization, and luteolysis occurs throughout the female's fertile lifespan. This Research Topic is mainly focused on the fascinating transformation of the hormone-producing theca (TC) and granulosa cells (GC) of the ovarian follicle during the formation, rescue or regression of the corpus luteum (CL). Articles of this Research Topic cover different mammalian species, novel cell culture models and approaches, and present data on a variety of specific factors and molecular mechanisms occurring within this dynamic tissue during its limited lifespan and the regulation of its primary hormonal product progesterone, which is critically important for female fertility. Bagnjuk and Mayerhofer review an interesting cell culture model of luteinized human GC derived from IVF patients. The authors emphasize how this model of a primate CL can be used to study two different forms of cell death, apoptosis, and necroptosis, which occur during luteolysis *in situ*. Future studies with these cells may identify novel molecular targets (e.g., blockers of necroptosis), which may provide insights into the regulation of luteal life and function. To study the biology of the feline CL, Hryciuk et al. established an approach to isolate small (SLC) and large (LLC) steroidogenic luteal cells from domestic cats. Using this cell culture model the authors studied the morphology and physiology of these individual cells; ultimately these studies may also provide insights into luteal biology of endangered feline species. In their review article, Abedel-Majed et al. discuss the lineage of the four most important hormone-producing cells of the bovine ovary, GC and TC before, and SLC and LLC after the preovulatory luteinizing hormone (LH) surge. Growth factors, androgen excess and inflammatory cytokines and their molecular mechanisms affecting ovulation and formation of the CL are also discussed. Better understanding of the profound transformation processes these cells undergo will improve our knowledge on causes of anovulation induced infertility. Interesting insight into the unique transitional gonadotropin independence of the canine CL and in particular prostaglandin actions are provided by Tavares Pereira et al. By inhibiting cyclooxygenase-2 (COX-2) activity *in situ* these authors examined the effects that loss of prostaglandins had on luteal transcriptomes at different post-ovulatory stages. With luteal maturation and emerging gonadotropin dependence, the canine CL transcriptome became more sensitive to COX-2 inhibition. These studies set the stage for more in depth functional examination of the mechanisms of prostaglandin-regulated cellular proliferation, immune cell infiltration and steroidogenesis. Likszo et al. examined the changing proteome during the folliculo-luteal transition in pigs. Proteins expressed in pre-ovulatory follicles were associated with cellular infiltration, endoplasmic stress responses and the protein ubiquitination pathway, whereas early luteal stage proteins were associated with steroid metabolism, cell death and survival, free radical scavenging, and protein ubiquitination pathways. Novel mechanisms of luteal cell differentiation, survival, and pathways regulating steroidogenesis in the newly formed CL were advanced by these authors. Lipid regulation of bovine luteal function was studied by Hughes et al.. In a targeted metabolomic analysis using ultra performance liquid chromatography-tandem mass spectrometry, several lipid mediators were identified as potential regulators of leukocyte activation, cell migration, and proliferation during early, mid and late luteal stages. Cultured luteal cells demonstrated a role of select lipid mediators in regulating luteal progesterone production and suggest a role for lipid mediators as regulators of steroidogenesis, immune cell activation and function, intracellular signaling, and cell survival and death. Two studies, Bender et al. and Walewska et al., demonstrate novel roles for thrombospondin 1 (THBS1) in follicular angiogenesis, luteinization, and ovulation in the primate CL, and in luteolysis of the equine CL, respectively. In the cynomologus macaque, Bender et al. block ovulation and follicular angiogenesis with an intrafollicular injection of anti-THBS1 antibody. Additionally, THBS1 treatment stimulated migration, proliferation, and sprout formation in cultured monkey ovarian microvascular endothelial cells. These groundbreaking studies clearly implicate THBS1 as an important regulator of ovulation and corpus luteum formation. Walewska et al. studied the interaction of THBS1 with hypoxia-inducible factor 1 alpha (HIF1α) and Nodal, a member of the transforming growth factor-beta superfamily in equine luteal tissue explants and demonstrated *in vitro* interactions between these factors. This study sheds light on the interactions between two novel regulators of luteal function. Berisha et al. examined bovine follicular fluid hormones and GC transcriptomics collected at different times following induced superovulation. The authors focus on prostaglandin family members and posit involvement in the local mechanisms regulating final follicle maturation and ovulation during the folliculo-luteal transition and formation of the CL. In their review, Teeli et al. describe the role of the proteasome/autophagy axis in the context of luteal formation and regression, respectively. The authors propose that CL regression may be governed by the ubiquitin-proteasome and autophagy pathways and consider the potential role of specific transcription factors involved in these events.

Taken together the articles in this Research Topic present novel data, approaches and ideas on the development of the corpus luteum, and its subsequent function and demise. However, in spite of these new perspectives and insights, which will provide useful stimuli for future approaches, our understanding of this essential female hormonal gland remains incomplete. Further efforts are necessary to further elucidate its most important key feature, its ephemeral nature.

## Author Contributions

JV, LC, and JP have written this editorial.

## Conflict of Interest

The authors declare that the research was conducted in the absence of any commercial or financial relationships that could be construed as a potential conflict of interest.

